# Comparison of Clinical Trajectories before Initiation of Renal Replacement Therapy between Diabetic Nephropathy and Nephrosclerosis on the KDIGO Guidelines Heat Map

**DOI:** 10.1155/2016/5374746

**Published:** 2015-12-29

**Authors:** Masanori Abe, Kazuyoshi Okada, Noriaki Maruyama, Hiroyuki Takashima, Osamu Oikawa, Masayoshi Soma

**Affiliations:** ^1^Division of Nephrology, Hypertension and Endocrinology, Department of Internal Medicine, Nihon University School of Medicine, Tokyo 173-8610, Japan; ^2^Division of General Medicine, Department of Internal Medicine, Nihon University School of Medicine, Tokyo 173-8610, Japan

## Abstract

This study investigated differences between the clinical trajectories of diabetic nephropathy and nephrosclerosis using the Kidney Disease: Improving Global Outcomes (KDIGO) heat map and the clinical characteristics between the two diseases at RRT initiation. This single-center, retrospective study enrolled 100 patients whose estimated glomerular filtration rate (eGFR) was ≥45 mL/min/1.73 m^2^ at their first visit and who were initiated on RRT. Fifty consecutive patients were assigned to each of the diabetic nephropathy and nephrosclerosis groups. All data for simultaneously measured eGFR and urinary albumin to creatinine ratio (UACR) were collected from first visit to RRT initiation and were plotted on the KDIGO heat map. Diabetic nephropathy was characterized by higher blood pressure and UACR and lower age, eGFR, and serum albumin levels compared with nephrosclerosis at RRT initiation. The vast majority of patients with diabetic nephropathy and eGFR < 60 mL/min/1.73 m^2^ had concomitant macroalbuminuria, whereas for patients with nephrosclerosis, even when eGFR was <45 mL/min/1.73 m^2^, many still had normoalbuminuria or microalbuminuria. The rate of decline of eGFR was significantly faster in the diabetic nephropathy group than that in the nephrosclerosis group. The clinical trajectories of diabetic nephropathy and nephrosclerosis differed markedly on the KDIGO heat map.

## 1. Background

Chronic kidney disease (CKD) progressively increases the risk of end-stage kidney disease (ESKD) and cardiovascular disease in line with its severity [1]. The prevalence of ESKD is expected to rise steeply over the next few decades, driven by population ageing and the increasing prevalence of diabetes and hypertension [2–4]. Although renal replacement therapy (RRT), via dialysis or renal transplantation, is a potentially lifesaving treatment for patients with ESKD, it is costly.

Type 2 diabetes mellitus is among the leading causes of CKD, including ESKD, in both developed and developing countries; in various countries including the USA and Japan, type 2 diabetes mellitus accounts for nearly 50% of patients on incident dialysis [2, 3]. In the USA in 2012, nephrosclerosis was the second most common primary disease after diabetic nephropathy [2], and in Japan in 2011, nephrosclerosis was the third most common primary disease (12.3%) after diabetic nephropathy and chronic glomerulonephritis [5]. In relation to the aging of new dialysis patients, the percentage of patients who had nephrosclerosis and were newly started on dialysis continuously increased. Since about 2000, the rate of increase in the annual number of new dialysis patients with chronic glomerulonephritis has been negative [5]. Therefore, in the future, nephrosclerosis will likely be the second most common primary disease in Japan as well as the USA. The management of diabetic nephropathy and nephrosclerosis is thus very important for helping prevent these patients from newly requiring RRT.

The Kidney Disease: Improving Global Outcomes (KDIGO) Clinical Practice Guideline for the Evaluation and Management of CKD was released in January 2013 [6]. KDIGO recommends CKD classifications based on cause, glomerular filtration rate (GFR) category, and albuminuria category. The cause of CKD is considered because it provides important prognostic information and influences treatment decisions. Albuminuria and estimated GFR (eGFR) provide independent information regarding the risk of CKD progression, cardiovascular disease, and mortality. In addition, clinicians and researchers are advised to categorize patients using a heat map generated by composite rankings of relative risk. However, differences between the clinical trajectories for diabetic nephropathy and nephrosclerosis, the two major primary causes of CKD, have not been revealed clearly on the KDIGO heat map.

Against this background, this retrospective study investigated differences between the clinical courses of diabetic nephropathy and nephrosclerosis using the KDIGO heat map and sought to determine how the clinical characteristics differed between the two diseases at the time of the RRT initiation.

## 2. Methods

This single-center, retrospective study, conducted between January 2011 and December 2013, was designed to compare the clinical courses of diabetic nephropathy and nephrosclerosis with respect to eGFR and the urinary albumin to creatinine ratio (UACR) in patients with CKD who were already receiving treatment from a nephrologist. Specifically, the study compared the clinical progression of the two diseases as represented by the heat map based on the prognosis of CKD by GFR and albuminuria category stated in the KDIGO 2012 Clinical Practice Guideline for the Evaluation and Management of Chronic Kidney Disease [6]. All data used in the analysis were collected from medical records. All study participants provided written informed consent, and the study protocol was approved by the Research Review Board of our University and conducted in accordance with the Declaration of Helsinki (Clinical Trial Registration Number: UMIN000017502).

Inclusion criteria were (1) patients who underwent RRT initiation at our hospital during January 2011 and December 2013 and (2) eGFR ≥ 45 mL/min/1.73 m^2^ at the first visit to our hospital. Exclusion criteria were (1) age < 20 years at RRT initiation, (2) RRT initiated due to acute kidney injury (AKI), and (3) primary cause of CKD other than diabetic nephropathy and nephrosclerosis (i.e., glomerulonephritis, cystic disease, or vasculitis). Diabetic nephropathy and nephrosclerosis were diagnosed by renal biopsy or medical history. Specifically, diabetic nephropathy was defined as diagnosis based on kidney biopsy (*n* = 15), on the presence of type 1 diabetes (*n* = 2), or on fulfillment of all the following criteria (*n* = 33): (1) diabetes duration ≥ 10 years; (2) clear presence of diabetic retinopathy; (3) no history of proteinuria or hematuria prior to the first visit; (4) other primary kidney disease, such as secondary, hereditary, cystic, or drug-induced kidney disease or vasculitis completely ruled out by blood work or imaging diagnostics. Nephrosclerosis was diagnosed by kidney biopsy (*n* = 10) or fulfillment of all of the following criteria (*n* = 40): (1) no history of comorbid diabetes prior to the first visit or during the observational period; (2) duration of hypertension ≥ 10 years; (3) no history of proteinuria or hematuria prior to the first visit; (4) presence of hypertensive retinopathy by fundus examination; and (5) other primary kidney disease, such as secondary, hereditary, cystic, drug-induced kidney disease, or vasculitis completely ruled out by blood work or imaging diagnostics. Subjects were assigned to either the diabetic nephropathy group or nephrosclerosis group at RRT initiation, with 50 consecutive subjects enrolled per group for a total of 100 subjects.

All data for simultaneously measured eGFR and UACR were used to monitor the clinical course from the first visit to RRT initiation and were collected from the medical records. These data were plotted on the heat map according to the KDIGO guidelines. Serum samples were assayed for creatinine (sCr) at a central laboratory (Central Laboratory; SRL Co., Tokyo, Japan) with the enzymatic Cr assay method using a Japan electron Cr auto-analyzer (JCA-BM8060; JEOL Ltd., Tokyo, Japan) and enzyme solution (Preauto-S CRE-L; Sekisui Medical Co., Ltd., Tokyo, Japan). To assess urinary albumin excretion, we measured urinary concentrations of albumin and Cr (albumin/Cr ratio) in spot urine samples. Urinary albumin was measured using the immunoturbidimetric assay. Glomerular filtration rate was estimated using the modified, final recommended equation for Japanese patients issued by the Japanese Society of Nephrology-CKD Initiatives, as eGFR values obtained by this method are more accurate for Japanese patients with CKD [7]. The formula was as follows:

(1) eGFR  mL/min  per  1.73 m2=194×sCr−1.094×age−0.287  × 0.739  for  women.

The composite ranking of relative risk by GFR and albuminuria levels was calculated according to the 2012 KDIGO guidelines using the following definitions: no CKD (green zone), G1A1 and G2A1; moderate risk (yellow zone), G1A2, G2A2, and G3aA1; high risk (orange zone), G1A3, G2A3, G3aA2, and G3bA1; and very high risk (red zone), G3aA3, G3bA2-3, all G4, and all G5 [6]. Blood pressure (BP) was measured at the outpatient clinic according to the Japanese Society of Hypertension 2009 guidelines [8]. Measurements were performed in duplicate every month using a sphygmomanometer (Nippon Colin, Tokyo, Japan) with the patient in a sitting position after a 5 min period of rest. Patients, particularly those with dietary restrictions, were given guidance on how to maintain their diet. Doses of antihypertensive agents, including angiotensin receptor blockers (ARBs), angiotensin-converting enzyme (ACE) inhibitors, calcium channel blockers, and diuretics, were adjusted during the study period to maintain the target BP level of <130/80 mmHg.

### 2.1. Statistical Analysis

Data were analyzed on the basis of assigned groups and are expressed as the mean ± SD or median [interquartile range], as appropriate. Continuous variables were compared using Student's *t*-test or the Mann-Whitney *U* test, and categorical variables were compared by the chi-square or Fisher's exact test as appropriate to the data distribution. To analyze the time course changes in eGFR, we fitted scatterplot smoothing curves to all the eGFR measures for all the patients in each group. Then, we used the Mann-Whitney *U* test to compare the eGFR decline (mL/min/1.73 m^2^ per year) between the groups. The eGFR time course data within groups were analyzed by repeated-measures analysis of variance (ANOVA), while changes between the two groups were analyzed by two-way ANOVA followed by Dunnett's test. To analyze the time course changes in albuminuria, we fitted scatterplot smoothing curves to all UACR measures for all the patients in each group. Then, we used the Mann-Whitney *U* test to compare the regression coefficients between the groups. Statistical significance was set at *P* < 0.05. All analyses were performed using JMP ver. 11 software (SAS Institute Ltd., Cary, NC, USA).

## 3. Results

### 3.1. Study Population and Characteristics at the First Visit and RRT Initiation

The clinical characteristics of the patients at the first visit are shown in Table 1. At the first visit, mean age was significantly higher in the nephrosclerosis group than in the diabetic nephropathy group. Body mass index (BMI) was significantly higher in the diabetic nephropathy group. Although there was no significant difference in heart rate, both systolic and diastolic BP was significantly higher in the diabetic nephropathy group. Serum Cr level was significantly lower and eGFR was significantly higher in the diabetic nephropathy group. There was no significant difference in hemoglobin or serum albumin level between the groups. Mean glycated hemoglobin level was 7.6 ± 0.6% in the diabetic nephropathy group.

The patients' clinical characteristics and medications being taken at RRT initiation are shown for each group in Table 2. Mean retrospective observational period and mean time of simultaneous measurement of eGFR and albuminuria did not significantly differ between the groups. At RRT initiation, mean age was significantly higher in the nephrosclerosis group than in the diabetic nephropathy group. BMI was significantly higher in the diabetic nephropathy group. Although there was no significant difference in heart rate, both systolic and diastolic BP was significantly higher in the diabetic nephropathy group. There was a significantly higher occurrence of cardiovascular comorbidity, in particular ischemic heart disease, in the diabetic nephropathy group. All patients had hypertension and were taking antihypertensive medication, with renin-angiotensin system (RAS) inhibitors including ARBs, ACE inhibitors, and direct renin inhibitors being the most common, followed by calcium channel blockers. Although 49 patients in the diabetic nephropathy group and 46 patients in the nephrosclerosis group had used diuretics, thiazide diuretics were used by only 3 patients in the diabetic nephropathy group and none in the nephrosclerosis group; other patients used loop diuretics. Although there was no significant difference in the type of antihypertensive agents used in the two groups, the number of such agents used per person was significantly greater in the diabetic nephropathy group.

### 3.2. Laboratory Data at RRT Initiation

The final data set collected before RRT initiation is shown in Table 3. The nephrosclerosis group had significantly higher serum Cr levels and lower eGFR values than the diabetic nephropathy group. The diabetic nephropathy group had a significantly higher UACR and a significantly lower serum albumin level. The diabetic nephropathy group had significantly higher triglyceride, N-terminal pro-brain natriuretic peptide (NT-proBNP), and C-reactive protein (CRP) levels and significantly lower high-density lipoprotein (HDL)-cholesterol levels. Hemoglobin level did not differ significantly between the groups. The glycated hemoglobin level was significantly decreased at RRT initiation compared with that at the first visit in the diabetic nephropathy group (*P* < 0.0001).

### 3.3. Time Course of eGFR Decline and Urinary Albumin Excretion Rate

**Figure 1:**
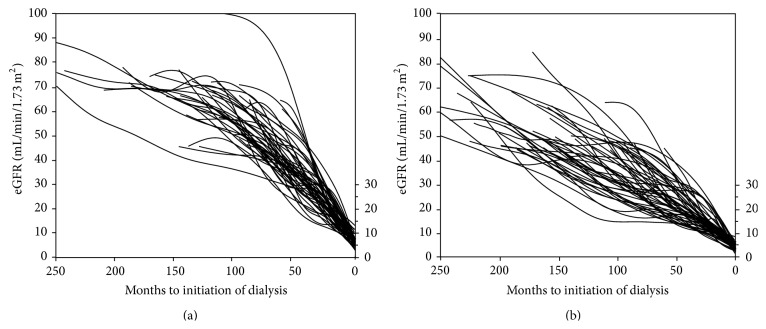
Estimated glomerular filtration rate (eGFR) trajectories with individual slopes during the observation period in the two groups. (a) Smoothing curve for the diabetic nephropathy group; (b) smoothing curve for the nephrosclerosis group.

**Figure 2:**
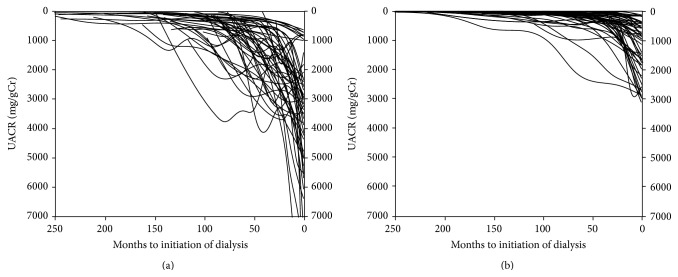
Urinary albumin to creatinine ratio (UACR) trajectories with individual slopes during the observation period in the two groups. (a) Smoothing curve for the diabetic nephropathy group; (b) smoothing curve for the nephrosclerosis group.

Figure 1 shows the eGFR trajectories of individuals in the diabetic nephropathy (a) and nephrosclerosis (b) groups. The mean eGFR slopes from first visit to RRT initiation for the diabetic nephropathy group and nephrosclerosis group were −6.6 ± 2.4 and −3.6 ± 1.2 mL/min/1.73 m^2^ per year, respectively (*P* < 0.0001). The duration between the observation of eGFR < 45 mL/min/1.73 m^2^ and RRT initiation was 59 ± 26 months in the diabetic nephropathy group and 94 ± 28 months in the nephrosclerosis group, showing a significant difference between the groups (*P* < 0.0001). Furthermore, the rates of decline in eGFR in the diabetic nephropathy group and nephrosclerosis group were −9.9 ± 5.3 and −4.8 ± 2.2 mL/min/1.73 m^2^ per year, respectively (*P* < 0.0001).  Figure 2 shows the UACR trajectories of individuals in the diabetic nephropathy (a) and nephrosclerosis (b) groups. The regression coefficient was −23.3 [−34 to −13] in the diabetic nephropathy group and −4.7 [−9.7 to −2.2] in the nephrosclerosis group, again showing a significant difference between the groups (*P* < 0.0001).

### 3.4. Clinical Course on the KDIGO Heat Map

**Figure 3:**
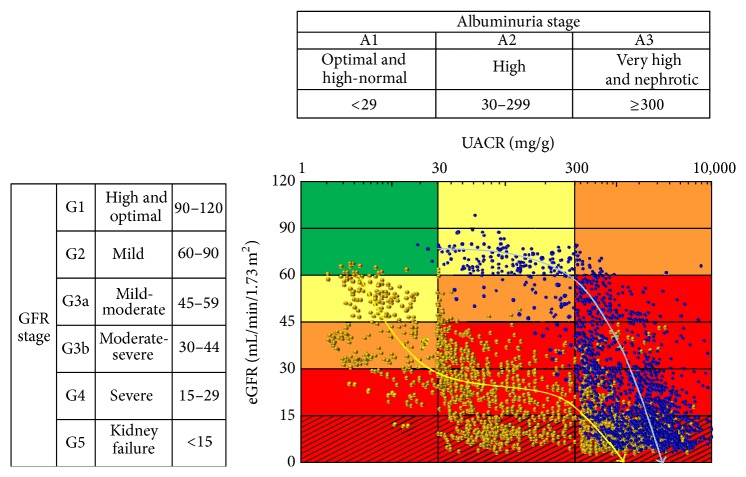
Data of simultaneously measured eGFR and UACR from the first visit to RRT initiation in the diabetic nephropathy group and nephrosclerosis group as represented on the KDIGO heat map. Blue and yellow bold arrows indicate clinical trajectories of the diabetic nephropathy and nephrosclerosis groups, respectively. eGFR, estimated glomerular filtration rate; GFR, glomerular filtration rate; KDIGO, Kidney Disease: Improving Global Outcomes; UACR, urinary albumin to creatinine ratio.

Figure 3 shows all plotted data for simultaneously measured eGFR and UACR from the first visit to our hospital to final data collection before RRT initiation in the two groups. In the diabetic nephropathy group, only 3 patients (6%) were plotted into the no CKD (G2A1) category at the first visit. Five patients (10%) and 2 patients (4%) were plotted into the high risk G3aA2 and G2A3 categories, respectively; the remaining 40 patients (80%) were plotted into the moderate risk category. In the nephrosclerosis group, 3 patients (6%) were plotted into the no CKD (G2A1) category at the first visit. However, 6 patients (12%) were plotted into the high risk category (G3aA2), and the remaining 41 patients (82%) were plotted into the moderate risk category (G3aA1). None of the patients in the nephrosclerosis group were plotted into G2A2 or G2A3, unlike many patients in the diabetic nephropathy group. In the diabetic nephropathy group, risk categories progressed from moderate or high risk to very high risk when GFR was reduced to <60 mL/min/1.73 m^2^. In other words, when eGFR was reduced to <60 mL/min/1.73 m^2^, albuminuria showed progression from the A2 to A3 stage. Moreover, the eGFR decline resulted in further elevation of albuminuria up to 3000 (interquartile range, 2084 to 4184) mg/gCr at RRT initiation. All cases underwent RRT initiation at the G5A3 stage. On the other hand, in the nephrosclerosis group, when eGFR was reduced to <45 mL/min/1.73 m^2^ and albuminuria had progressed from the A1 to A2 stage, the risk category changed from moderate or high risk to very high risk. Thereafter, eGFR gradually decreased and albuminuria gradually increased. Eight patients (16%) were started on RRT while remaining at the A2 stage, whereas the other 42 patients (84%) had progressed to A3 at RRT initiation.

## 4. Discussion

These results reveal that the clinical courses of the two primary causative diseases of CKD—diabetic nephropathy and nephrosclerosis—differ considerably when represented on the KDIGO heat map. As shown in Figure 3, diabetic nephropathy and nephrosclerosis showed contrasting characteristic courses. Furthermore, there was a significant difference in the rate of decline of eGFR between patients with diabetic nephropathy and those with nephrosclerosis. This indicates that even when eGFR levels are comparable, the subsequent progression in diabetic nephropathy would be more rapid compared with that for nephrosclerosis. It is recommended that patients be referred to nephrology at stage 4 CKD to prepare for RRT. Nephrologists and general physicians should be aware of the different clinical courses of these two CKDs and should aim to differentiate the causes of CKD upon physician examination.

Declines in eGFR, such as a 30% reduction over 2 years, were reported to be strongly and consistently associated with the risks for ESKD and mortality and have been considered an alternative endpoint for CKD progression [9]. Although the traditional view of kidney function decline in CKD is a steady linear decline (or slope), albeit at different rates among individuals, recent studies have evaluated the trajectories of decline and have shown they are often not linear [10–12]. The average overall rate of decline reported in 1441 adult individuals with stage 3–5 CKD was 1.47 mL/min/1.73 m^2^; however, the rate was faster in individuals with eGFR < 30 mL/min/1.73 m^2^ and accelerated in the year before the development of ESKD [10]. Individuals with steeper trajectories were more likely to have been hospitalized and to receive a diagnosis of AKI during hospitalization [12]. Although the patients who began RRT due to AKI were not included in the present study, diabetic nephropathy might be predisposed to rapid decline compared with nephrosclerosis. These findings highlight the heterogeneity of the rates of decline of eGFR and should lead to more individualized approaches to preparation for ESKD and transplant referral. Further studies should focus on identifying risk factors for the rapid decline of eGFR to allow for more timely intervention, as trajectories according to the primary disease of CKD have not been considered in previous studies.

Recently, the prognostic significance of identifying individuals with diabetes and an early decline (starting at <60 mL/min/1.73 m^2^) in GFR (>3.5 mL/min/1.73 m^2^ per year) that is over and above what would be expected with aging alone has been highlighted, with this early decline being linked to the development of ESKD in type 1 diabetes [13, 14]. Krolewski reported that 25% of patients with diabetes can be considered to have rapid progressive renal decline (eGFR slope < −7 mL/min/year) and these patients progressed to ESKD within 2–10 years. Another 25% showed moderate progressive renal decline (eGFR slope −7 to −3 mL/min/year), and most of them progressed or will progress to ESKD within 10–30 years. The remaining patients (50%) will have slow or no progressive renal decline and few may progress to ESKD during 30 years of follow-up [15]. Furthermore, the prevalence rate of patients showing such renal decline is 10%, 32%, and 50% among patients with normoalbuminuria, microalbuminuria, and proteinuria, respectively [16]. In the present study, the GFR decline rate in the diabetic nephropathy group was −6.6 mL/min/1.73 m^2^ per year during 115 months overall. However, when the eGFR declined to less than 45 mL/min/1.73 m^2^, the decline rate accelerated to −9.9 mL/min/1.73 m^2^ per year.

However, some studies have shown microalbuminuria remission rates of 21–64% in patients with diabetes [17–22]. These high rates of microalbuminuria remission have been linked to the use of RAS inhibitors in some studies. Currently, there is known to be a four- to fivefold magnitude increase in the risk for ESKD in patients with type 1 diabetes or type 2 diabetes and microalbuminuria [23]. However, many of our patients with ESKD due to diabetic nephropathy had resistant hypertension and higher blood pressures compared with those with ESKD due to nephrosclerosis, despite taking significantly larger numbers of antihypertensive agents. In the absence of antihypertensive therapy, GFR may decrease by 10–15 mL/min per year during stage 4, which is characterized by clinically detectable proteinuria, hypertension, and declining GFR [24]. Therefore, our data demonstrate that, in patients with diabetic nephropathy, the inhibition of progression from microalbuminuria to macroalbuminuria is important for preventing progression to ESKD. Although RAS inhibitors remain the cornerstone of therapy, the management of patients who do not respond to them remains an issue.

Recent studies demonstrated that normoalbuminuric renal insufficiency is not uncommon for diabetic patients, especially those with type 2 diabetes [25]. There are several possible pathogenic mechanisms that may account for the development of normoalbuminuric renal insufficiency. Renal ischemia due to intrarenal arteriosclerosis and disproportionately advanced tubulointerstitial lesions, despite minor diabetic glomerular lesions, which denote the presence of diabetic kidney lesions as well as nephrosclerosis, are likely to be related to the development of normoalbuminuric renal insufficiency [26, 27]. The clinical characteristics of such patients include older age, female predilection, shorter duration of diabetes, lower prevalence of hypertension, smoking, previous cardiovascular disease, and antihypertensive agents including RAS inhibitors, lower levels of glycated hemoglobin, and higher levels of HDL-cholesterol [28–30]. The diabetic nephropathy group in the present study included patients in whom diabetic nephropathy showed a typical clinical course, since none had normoalbuminuric renal insufficiency.

We recognize that our study is limited by the diagnostic methods used for diabetic nephropathy and nephrosclerosis. Moreover, there were only 17 and 10 biopsy-proven patients in the diabetic nephropathy and nephrosclerosis groups, respectively. Therefore, patients with chronic glomerulonephritis might have been included in the diabetic nephropathy group. However, all patients in the diabetic nephropathy group had diabetic retinopathy and a prolonged duration of diabetes. Therefore, we believe that the diagnosis of diabetic nephropathy was fairly certain in our patients. A second limitation is that the frequency of simultaneous measurement of eGFR and UACR was lower for the duration from the first visit to an eGFR of 45 mL/min/1.73 m^2^, because in Japan, many patients are commonly followed by a general physician. Patients were thereafter treated by a nephrologist only when eGFR decreased to <45–30 mL/min/1.73 m^2^. Therefore, there was less data for the duration in the moderate and high risk categories than for the very high risk category, and the precise duration from the no CKD to moderate risk categories (G2A2 and G3aA1) could not be determined. Although some patients with diabetic nephropathy rapidly progress to ESKD, these patients were excluded from the present analysis because they had less data available for simultaneous measurements and most of them already had eGFR < 30 mL/min/1.73 m^2^ at the first visit. Moreover, we could not clarify the eGFR and UACR trajectories of patients who had no or minimal decline in eGFR over the study period, since our study design allowed for only the investigation of subjects that ultimately progressed to ESKD. Lastly, the sample size was relatively small, and our study was retrospective. However, if this study were to be performed as a prospective study, we would need a relatively long period to complete it, as the endpoint of the study is RRT initiation. Nevertheless, additional studies are necessary to more firmly establish whether the risk categories of the KDIGO classification precisely reflect prognosis; not only the requirements for RRT but also cardiovascular events should be considered as endpoints since the risk categories of the KDIGO classification have three distinct indications, namely, risks for ESKD, cardiovascular events, and all-cause mortality.

## 5. Conclusions

This retrospective analysis showed that the clinical trajectories to RRT initiation on the KDIGO heat map differed between diabetic nephropathy and nephrosclerosis. The rate of decline of eGFR in the diabetic nephropathy group was significantly faster than that in the nephrosclerosis group. Therefore, identification of the primary disease of CKD by kidney biopsy might be important for determining the likelihood of progression to ESKD. Furthermore, compared with nephrosclerosis, diabetic nephropathy was characterized at RRT initiation by higher BMI, higher systolic and diastolic BPs, and higher CRP, NT-proBNP, and albuminuria levels as well as lower age and serum albumin levels. Further studies are needed to clarify the factors that influence the progression to ESKD.

## Figures and Tables

**Table 1:** Clinical characteristics at the first visit in the two groups.

	Diabetic nephropathy	Nephrosclerosis	*P* value
*n* (male/female)	50 (34/16)	50 (35/15)	0.833
Age (years)	57.1 ± 9.2	65.6 ± 8.4	<0.0001
Body mass index (kg/m^2^)	24.7 ± 2.5	22.5 ± 1.7	<0.0001
Systolic blood pressure (mmHg)	149 ± 8	146 ± 10	0.038
Diastolic blood pressure (mmHg)	86 ± 7	82 ± 10	0.012
Heart rate (bpm)	76 ± 8	76 ± 7	0.989
Serum creatinine (mg/dL)	0.9 ± 0.2	1.1 ± 0.2	<0.0001
eGFR (mL/min/1.73 m^2^)	65.6 ± 10.5	50.0 ± 6.3	<0.0001
UACR (mg/gCr)	131 [57, 189]	25 [15, 31]	<0.0001
Hemoglobin (g/dL)	13.7 ± 0.9	13.5 ± 0.7	0.129
Serum albumin (g/dL)	4.0 ± 0.3	4.0 ± 0.2	0.580
Type of diabetes (type 1/2)	2/48	—	—
Glycated hemoglobin (%)	7.6 ± 0.6	5.4 ± 0.3	<0.00001

Data are expressed as mean ± SD, median [interquartile range], or *n*.

GFR, glomerular filtration rate; UACR, urinary albumin to creatinine ratio.

**Table 2:** Clinical characteristics and medications at the initiation of RRT in the two groups.

	Diabetic nephropathy	Nephrosclerosis	*P* value
*n* (male/female)	50 (34/16)	50 (35/15)	0.833
Age (years)	67.2 ± 9.6	78.8 ± 6.4	<0.0001
Observational periods (months)	115 ± 57	122 ± 35	0.447
Measurement times (/year)	4.4 ± 2.5	4.4 ± 2.6	0.955
Measurement times (/person)	34 ± 11	37 ± 10	0.109
Body mass index (kg/m^2^)	24.8 ± 2.5	22.1 ± 1.7	<0.0001
Systolic blood pressure (mmHg)	147 ± 15	137 ± 9	<0.0001
Diastolic blood pressure (mmHg)	80 ± 12	73 ± 10	0.0003
Heart rate (bpm)	77 ± 7	76 ± 8	0.541
Mode of renal replacement therapy % (*n*)			
Hemodialysis	92 (46)	92 (46)	—
Peritoneal dialysis	8 (4)	8 (4)	—
Kidney transplantation	0 (0)	0 (0)	—
Cardiovascular comorbidities % (*n*)	34 (17)	18 (9)	0.069
Ischemic heart disease	28 (14)	12 (6)	0.046
Cerebrovascular disease	6 (3)	4 (2)	0.650
Peripheral artery disease	4 (2)	2 (1)	0.562
Diabetic retinopathy % (*n*)	100 (50)	—	—
*Medication %* (*n*)			
Antihypertensive agents			
Angiotensin receptor blockers	98 (49)	90 (45)	0.093
Angiotensin-converting enzyme inhibitors	14 (7)	4 (2)	0.082
Direct renin inhibitors	8 (4)	2 (1)	0.173
Calcium channel blockers	98 (49)	94 (47)	0.312
Diuretics	98 (49)	92 (46)	0.172
*β*-blockers	30 (15)	14 (7)	0.054
*α*-blockers	30 (15)	14 (7)	0.054
Number of antihypertensive agents (per person)	3.76 ± 0.2	3.10 ± 0.1	0.0006
Antidiabetic agents			
Insulin	38 (19)	—	—
Oral hypoglycemic agents	58 (29)	—	—
Diet therapy alone	4 (2)	—	—
Erythropoiesis stimulating agents	100 (50)	98 (49)	0.319
Statins	80 (40)	78 (39)	0.808
Active vitamin D	94 (47)	92 (46)	0.698

Data are expressed as mean ± SD, %, or *n*.

**Table 3:** Laboratory data before the initiation of RRT in the two groups.

Variables	Diabetic nephropathy	Nephrosclerosis	*P* value
Serum creatinine (mg/dL)	8.8 ± 1.4	9.9 ± 1.6	0.004
eGFR (mL/min/1.73 m^2^)	5.5 ± 1.1	4.2 ± 0.8	<0.0001
UACR (mg/gCr)	3000 [2084, 4184]	972 [490, 1830]	<0.0001
Hemoglobin (g/dL)	10.1 ± 0.9	10.7 ± 0.9	0.290
Serum albumin (g/dL)	3.3 ± 0.6	3.7 ± 0.4	0.0012
Total cholesterol (mg/dL)	173 ± 38	167 ± 35	0.341
HDL-cholesterol (mg/dL)	45 ± 13	52 ± 13	0.013
Triglyceride (mg/dL)	141 [96, 178]	94 [76, 127]	<0.0001
Glycated hemoglobin (%)	6.5 ± 0.7	5.6 ± 0.4	<0.0001
NT-proBNP (pg/mL)	2670 [1531, 7209]	1298 [594, 3226]	0.021
C-reactive protein (mg/dL)	0.17 [0.10, 0.29]	0.09 [0.03, 0.14]	<0.0001

Data are expressed as mean ± SD or median [interquartile range]. eGFR, estimated glomerular filtration rate; HDL, high-density lipoprotein; NT-proBNP, N-terminal pro-brain natriuretic peptide; UACR, urinary albumin to creatinine ratio.
